# Perceived stress during labor and its association with depressive symptomatology, anxiety, and pain catastrophizing

**DOI:** 10.1038/s41598-021-96620-0

**Published:** 2021-08-20

**Authors:** Hon Sen Tan, T. Agarthesh, Chin Wen Tan, Rehena Sultana, Helen Yu Chen, Tze-Ern Chua, Ban Leong Sng

**Affiliations:** 1 Department of Women’s Anesthesia, KK Women’s and Children’s Hospital, Singapore, Singapore; 2 Anesthesiology and Perioperative Sciences Academic Clinical Program, Duke-NUS Medical School, Singapore, Singapore; 3 Centre for Quantitative Medicine, Duke-NUS Medical School, Singapore, Singapore; 4 Department of Psychological Medicine, KK Women’s and Children’s Hospital, Singapore, Singapore; 5 Pediatrics Academic Clinical Program, Duke-NUS Medical School, 100 Bukit Timah Road, Singapore, 229899 Singapore

**Keywords:** Psychology, Medical research

## Abstract

Perceived stress is a dimension of the maternal stress response, however little data is available on perceived stress levels and its associated psychological risk factors during labor. In this secondary data analysis from a prospective study evaluating epidural regimens, we investigated the potential associations between depressive symptomatology, anxiety, and pain catastrophizing with perceived stress during labor. Healthy nulliparous adult women with term singleton pregnancies requesting for epidural analgesia in early labor were included. Assessments were administered after epidural analgesia and adequate pain relief were achieved. Perceived stress (Perceived Stress Scale, PSS, high PSS ≥ 16), depressive symptomatology (Edinburgh Postnatal Depression Scale, EPDS, high EPDS ≥ 10), and pain catastrophizing (Pain Catastrophizing Scale, PCS, high total PCS ≥ 25) were assessed as categorical variables. Additionally, anxiety (State-trait Anxiety Inventory, STAI), PCS total and its subscales (rumination, magnification and helplessness) were analyzed as continuous variables. Univariate and multivariable logistic regression models were used to identify factors associated with high PSS. Of 801 women included, 411 (51.9%) had high PSS. High EPDS (OR 2.16, 95%CI 1.36–3.44), increasing trait anxiety (OR 1.17, 95%CI 1.14–1.20), and increasing pain magnification (OR 1.12, 95%CI 1.05–1.19) were independently associated with high PSS. Depressive symptomatology, trait anxiety, and pain magnification were associated with perceived stress during labor, providing impetus for future research aimed at detecting and alleviating stress and its psychological or pain association factors.

## Introduction

The processes of labor and childbirth involve a multitude of psychological and physical demands that result in maternal stress. Although the physiological manifestations of stress, including neuroendocrine responses and elevated stress biomarkers such as norepinephrine, epinephrine, and cortisol are well elucidated^1^, the substantial inter-individual variation in the levels of these stress biomarkers^1^ underscores the multifaceted and complex influence of environmental (e.g. prior stressful life events) and psychological (e.g. anxiety or depression) risk factors contributing to the maternal stress response during labor^2,3^. In particular, psychological and environmental association factors comprise a dimension of the stress response known as perceived stress, which have been described as “the degree to which situations in one’s life are appraised as stressful”^2^.

Failure to alleviate perceived stress during pregnancy may have adverse maternal and neonatal consequences. Elevated levels of perceived stress during the antenatal period are associated with increased risk of gestational diabetes^4^, postnatal stress^5^, pre-term birth^6–8^, low fetal birth weight^9^, and lower childhood intelligence scores^10^. After childbirth, elevated perceived stress levels have demonstrated positive correlation with maternal depressive symptomatology and anxiety^11^.

Notably, the above studies have focused on two periods: early pregnancy and after childbirth, with limited available data regarding perceived stress during labor and delivery. High levels of stress during labor have been associated with deleterious effects including immunosuppression, delayed wound healing, depression, reduced uterine contractions, and prolonged labor^12^.

Given that maternal stress levels were shown to peak during labor^1^, this period may represent an important opportunity to reduce stress and associated adverse outcomes by targeting factors contributing to the stress response. For instance, the provision of labor epidural analgesia has been shown to reduce physiological stress and decrease plasma levels of norepinephrine and epinephrine^1^. However, labor epidural analgesia does not directly address co-existing psychological and pain association factors that may contribute to perceived stress, such as pain catastrophizing, anxiety, and depression^13^. Although these association factors exhibit similar temporal patterns as the maternal stress response and peak towards the end of pregnancy^13^, the potential associations with perceived stress during labor have yet been investigated. This represents an important knowledge gap as available evidence suggests that maternal stress during labor arise from specific factors occurring during this period. These include the fear of labor pain or episiotomy, as well as anxiety and fear regarding her inability to give birth, dying during childbirth, and lack of healthcare support^14^. Hence, knowledge of psychological distress and pain association factors contributing to maternal stressors during labor may facilitate and justify targeted pre-emptive management with the aim of reducing maternal perceived stress during labor and delivery.

Therefore, the objectives of this study were to determine if perceived stress were associated with depressive symptomatology, anxiety, and pain catastrophizing in laboring mothers receiving epidural analgesia.

## Methods

This was a secondary analysis of data from a prospective study that evaluated epidural delivery regimens for labor analgesia. The study received ethics approval from SingHealth Centralized Institutional Review Board (2014/670/D; 2018/3128), was registered on Clinicaltrials.gov (NCT02278601; date of registration 26/10/2014), and written informed consent was obtained from all study participants. This study protocol was developed based on the Strengthening the Reporting of Observational studies in Epidemiology (STROBE) guidelines.

The study population included healthy (American Society of Anesthesiologists (ASA) physical status II), nulliparous women aged 20–46 years, with singleton pregnancies over 36 weeks’ gestation, and who requested for epidural analgesia in early labor (cervical dilation ≤ 5 cm). We excluded women with multiple pregnancies, non-cephalic fetal presentation, obstetric and poorly controlled comorbidities, contraindications to neuraxial blockade, receipt of parenteral opioids within 2 h prior to epidural analgesia, and who had inadvertent dural puncture.

Perceived stress, depressive symptomatology, anxiety, and pain catastrophizing were assessed using validated questionnaires administered after initiation of labor epidural analgesia when the women were comfortable. The questionnaires used were the Perceived Stress Scale (PSS), Edinburgh Postnatal Depression Scale (EPDS), State-Trait Anxiety Inventory (STAI) and Pain Catastrophizing Scale (PCS), respectively. PSS, EPDS, and PCS were analyzed as categorical variables to facilitate generalizability and comparison with other studies utilizing these assessment tools. Justification for the threshold values used in this study are provided below.

### Perceived stress scale-10 (PSS-10)

The level of perceived stress was determined using the PSS-10, comprising 10 items graded on a five-point Likert scale, with a total possible score of 0–40. Perceived stress was studied as a categorical variable. Similar to other studies^10,15–17^, the median-split approach was used to determine the threshold of high versus low PSS scores. In our study cohort, women with PSS ≥ 16 (high PSS) were considered to have high levels of perceived stress, compared to those with PSS < 16 (low PSS). The validity and reliability of the PSS-10 have been previously demonstrated^18^.

### Edinburgh postnatal depression scale (EPDS)

The EPDS is a commonly used modality for assessing depressive symptomatology during pregnancy and up to a year postpartum. The EPDS comprises of 10 items, each with a four-point Likert scale from zero to three, and a total score ranging from 0–30. EPDS was analyzed as a categorical variable (high EPDS: EPDS ≥ 10 versus low EPDS: EPDS < 10), with the threshold selected based on the results of a systematic review^19^.

### State-trait anxiety inventory (STAI)

Maternal anxiety during labor was assessed using the STAI, which includes two subscales; the state anxiety scale measures the current state of anxiety, while the trait anxiety scale measures general and long-standing anxiety^20^. Each subscale is comprised of 20 items on a 4-point Likert scale from one to four, with total score ranging from 20–80. The levels of anxiety assessed by both state and trait anxiety scores were analyzed as continuous variables. The STAI has previously demonstrated good validity and reliability in obstetric patients^21^.

### Pain catastrophizing scale (PCS)

Pain catastrophizing was evaluated using the PCS, which contains three subscales assessing multiple dimensions of the catastrophizing construct: rumination, magnification and helplessness^22^. The PCS is a 13-item questionnaire on a five-point Likert scale from zero to four, with the total PCS score ranging from 0 to 52. In our study, PCS subscales and total PCS scores were analyzed as continuous variables. Additionally, total PCS was analyzed as a categorical variable (high PCS: PCS ≥ 25 versus low PCS: PCS < 25), based on recent study on PCS reporting in women receiving labor epidural analgesia and the recommendation of developer of PCS on the use of cut-off scores for clinical relevance of pain catastrophizing^23,24^.

### Statistical analysis

The primary outcome variable was analyzed as binary data with categories of high or low PSS. Maternal and relevant clinical characteristics were summarized according to PSS status. Categorical variables were presented as frequency with proportion, while continuous variables were presented as mean ± standard deviations (SD).

Univariate and multivariable logistic regression models were used to identify variables associated with high PSS. Quantitative association from the above models were reported as odds ratios (OR) with corresponding 95% confidence intervals (95% CI). Variables with -value < 0.2 from the univariate regression analysis and clinically meaningful variables were selected for the multivariable logistic regression analysis. Stepwise variable selection method was employed with selection significance levels to enter and stay of 0.2 and 0.05, respectively, to finalize the multivariable model. Area under the receiver operator characteristic (AUROC) curve was reported. Statistical significance was set at p-value < 0.05 and all tests were two-tailed. Data analysis was performed using SAS version 9.4 software (SAS institute, Cary, NC, USA).

## Results

**Figure 1:**
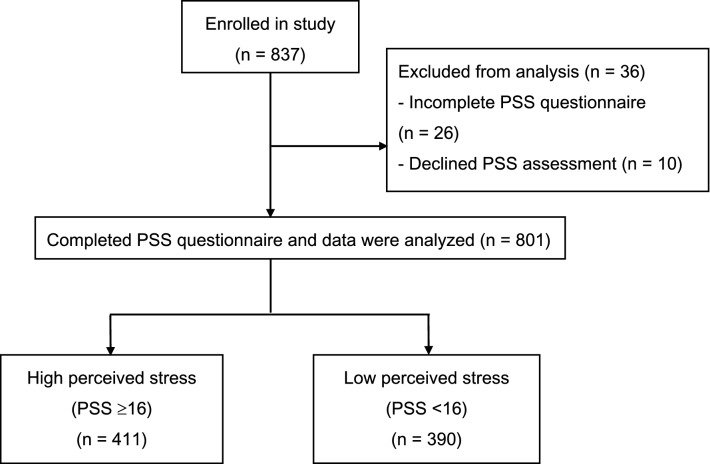
Study flowchart. Validated assessments were administered after initiation of epidural analgesia, with the patient comfortable.

Out of the 837 women enrolled between January 2015 and March 2019, we excluded 36 women who had incomplete PSS questionnaires or who declined the PSS assessment. Of the 801 women analyzed, 411 (51.9%) were assessed to have high PSS (Fig. 1). Pain scores (numerical rating scale, 0: no pain, 10: worst pain imaginable) prior to epidural placement (high PSS: 5.89 ± 3.17; low PSS: 5.56 ± 3.21) and after initiation of epidural analgesia (high PSS: 0.19 ± 0.91; low PSS: 0.16 ± 0.65) were similar between the groups. Univariate associations of high PSS with baseline maternal and clinical characteristics are presented in Table 1, with younger women (OR 0.95, 95%CI 0.92–0.98) and Malay race (OR 1.92, 95%CI 1.29–2.86) associated with high PSS. Based on univariate regression analysis, no other baseline maternal and clinical characteristics was associated with high PSS.

**Table 1:** Univariate associations of baseline parturient and clinical characteristics with perceived stress levels during labor.

Variable	High PSS (PSS ≥ 16) N = 411	Low PSS (PSS < 16) N = 390	Univariate associations	
			Unadjusted OR (95%CI)	*p* value
Age (years)	29.3 ± 4.4	30.2 ± 4.1	0.95 (0.92–0.98)	0.002
Race				0.010^a^
Chinese	226 (55.0)	237 (60.8)	Reference	–
Indian	43 (10.5)	50 (12.8)	0.90 (0.58–1.41)	0.650
Malay	86 (20.9)	47 (12.1)	1.92 (1.29–2.86)	0.001
Other	56 (13.6)	56 (14.4)	1.05 (0.69–1.59)	0.822
Height (cm)	159.4 ± 6.1	159.7 ± 5.7	0.99 (0.97–1.01)	0.417
Weight (kg)	69.9 ± 11.9	69.7 ± 10.6	1.00 (0.99–1.01)	0.818
Body mass index (kg.m^−2^)	27.6 ± 5.0	27.4 ± 4.1	1.01 (0.98–1.04)	0.523
Gestation (weeks)	37.7 ± 2.0	37.7 ± 2.1	0.99 (0.92–1.06)	0.689
Gravida	1.2 ± 0.6	1.2 ± 0.6	1.02 (0.79–1.31)	0.886
Analgesia prior to labor epidural	227 (55.2)	185 (47.4)	1.51 (0.58–3.92)	0.403

Data presented as n (%) or mean ± SD for categorical and continuous data respectively.

^a^Type 3 or omnibus -value.

Univariate and multivariable associations of depressive symptomatology, anxiety, and pain catastrophizing with high PSS are summarized in Table 2. Based on univariate analysis, high PSS was associated with increasing PCS rumination, magnification, helplessness, and total scores, STAI state and trait anxiety scores, as well as high EPDS. The final multivariable model showed that high EPDS (OR 2.16, 95%CI 1.36–3.44), increasing STAI trait anxiety score (OR 1.17, 95%CI 1.14–1.20), and increasing PCS magnification score (OR 1.12, 95%CI 1.05–1.19) were independently associated with high PSS, with an AUROC of 0.84.

**Table 2:** Univariate and multivariable associations of depressive symptomatology, anxiety, and pain catastrophizing with perceived stress levels during labor.

Variable	High PSS (PSS ≥ 16) N = 411	Low PSS (PSS < 16) N = 390	Univariate associations		Multivariable associations	
			Unadjusted OR (95%CI)	*P* value	Adjusted OR (95%CI)	*P* value
High EPDS^a^ (EPDS ≥ 10)	180 (43.8)	35 (9.0)	7.95 (5.34–11.84)	< 0.001	2.16 (1.36–3.44)	0.001
**STAI score**						
State anxiety	40.6 ± 9.4	32.8 ± 8.6	1.10 (1.08–1.12)	< 0.001		
Trait anxiety	41.5 ± 7.7	31.7 ± 6.7	1.20 (1.17–1.24)	< 0.001	1.17 (1.14–1.20)	< 0.001
**PCS score**						
Rumination	9.3 ± 4.4	7.3 ± 4.5	1.10 (1.07–1.14)	< 0.001		
Magnification	5.4 ± 3.0	3.5 ± 2.6	1.27 (1.20–1.34)	< 0.001	1.12 (1.05–1.19)	< 0.001
Helplessness	10.8 ± 6.0	7.9 ± 5.6	1.09 (1.06–1.12)	< 0.001		
High total PCS^b^ (PCS ≥ 25)	25.4 (12.2)	18.8 (11.5)	2.47 (1.85–3.30)	< 0.001		

Data presented as n (%) or mean ± SD for categorical and continuous data respectively.

^a^EPDS was analyzed as a categorical variable.

^b^Total PCS was analyzed as a categorical variable.

## Discussion

Our results demonstrate that the presence of depressive symptomatology, increasing trait anxiety, and increasing pain magnification were independently associated with elevated perceived stress levels in laboring women receiving epidural analgesia.

The association between increasing pain magnification and perceived stress during labor is novel and to our knowledge has yet been reported in prior studies. Pain magnification is one of three dimensions of the catastrophizing construct, which can be described as heightened attention to pain stimuli and inability to disengage from this state^25^. The appraisal theory based on Lazaraus and Folkman’s transactional model of stress and coping posits that this heightened attention stems from excessive focus on pain, in addition to evaluation of the painful stimulus as being extremely threatening^25^. Hence, it is possible that increasing pain magnification results in a disproportionate amount of attention and focus on actual or anticipated pain associated with labor, which may in turn manifest as elevated levels of perceived stress. Although the mechanism underlying this association between pain magnification and perceived stress should be investigated, our finding highlights the importance of managing maternal pain perceptions and expectations prior to labor.

Our finding that 26.8% of our cohort exhibited depressive symptomatology is consistent with the prevalence of 20% reported by another study of peripartum women in Singapore^26^. Moreover, we found that the presence of depressive symptomatology and increasing trait anxiety were associated with perceived stress during labor. These findings concur with that of Razurel et al., who postulated that elevated maternal stress reduces self-efficacy, which in turn exacerbates psychological pathologies and impairs adaptation to the parental role^11^. Additionally, increasing trait anxiety may act as a risk factor predisposing towards high stress levels and depression^27^. Regardless, the significant consequences of anxiety and depression during pregnancy, which include low birth weight^28^, pre-eclampsia, cesarean delivery, impaired maternal-neonatal interaction, and reduced feeling of parental self-efficacy^11,28,29^ underscore the importance of screening and management of these psychological association factors early in pregnancy.

The results of our study demonstrate the link between psychological health and stress during labor, thereby highlighting the importance of implementing appropriate measures to reduce maternal stress and associated factors. Importantly, the multifaceted nature of the maternal stress response suggests that a similarly multi-dimensional management approach is required to effectively ameliorate stress. Although the provision of optimal labor analgesia has been a clinical and research focus for several decades^30^, and was shown to reduce the physiological stress response^1^, the interplay between psychological factors and maternal stress during labor is not widely recognized. The postulation that perceived stress is an adaptive process stemming from maternal perception that an adverse event is insurmountable suggests that the provision of adequate emotional support during the peripartum period may be effective in alleviating stress^11^. Indeed, maternal satisfaction with support received from her parents, spouse, and the healthcare team has been shown to protect and moderate perceived stress and psychological health via a mechanism termed the “stress buffering model”^11^. Additionally, promptly addressing maternal stress during labor helps alleviate the fear of childbirth^31^.

Favorable healthcare-related outcomes are often achieved in settings such as Singapore, where the vast majority of women are managed by a specialist-led team. However, physical needs are often more emphasized, which though crucial, may neglect maternal emotional requirements with implications on mental health outcomes and birthing experience. The finding that over half of our cohort had high perceived stress scores despite uncomplicated obstetric histories and easy access to analgesia indicates the magnitude of their emotional needs. This study hopes to shed light on the interplay between physical and psychological (stress, depression and anxiety) aspects of childbirth, and highlights the need to provide emotional support. Future research should focus on identifying maternal perceived stress and psychological or pain association factors that would benefit from pre-emptive clinical management, in addition to the impact of psychological health and pain intensity during labor on postpartum outcomes.

We acknowledge several limitations to our study. Although we accounted for several baseline maternal and clinical characteristics, data on factors previously associated with perceived stress such as education, marital status, occupation, health-related quality of life, and intention for pregnancy and antenatal care were not collected or analyzed^32^. Additionally, potential confounders for psychological morbidity including baseline psychiatric and chronic pain conditions, smoking status, and alcohol consumption were not taken into account^33^. In this study, we sought to minimize the influence of labor pain on the maternal stress response by providing epidural analgesia, thereby focusing on determining the psychological risk factors associated with perceived stress. Hence, our results should be validated in women receiving other forms of labor pain relief. Furthermore, this study was performed in a single tertiary women’s and children’s hospital, which could have influenced the perception of healthcare support. Finally, our study cohort was predominantly comprised of multi-ethnic Asian women, who provided a multicultural scope to our research but limits the generalizability of our findings to other populations.

In conclusion, this study demonstrates that the presence of depressive symptomatology, as well as increasing trait anxiety and pain magnification were associated with maternal perceived stress during labor. In highlighting the interplay between physical and psychological aspects of childbirth, our findings advocate for routine pre-labor screening of perceived stress levels and the presence of psychological and pain association factors. Particular attention to the provision of pain relief and emotional support should be emphasized in high-risk women. These findings provide further impetus for future research into early detection and intervention aimed at alleviating stress and its associated factors during labor and delivery.

## Data Availability

The datasets generated and analyzed in this work are available for anyone who wishes to access the data by contacting the corresponding author.

## Abbreviations

ASA: American Society of Anesthesiologists
AUROC: Area under the receiver operator characteristic curve
CI: Confidence interval
COLEUS: Collaborative outcomes with labor epidural use study
EPDS: Edinburgh postnatal depression scale
OR: Odds ratio
PCS: Pain catastrophizing scale
PSS: Perceived stress scale
SD: Standard deviation
STAI: State-trait anxiety inventory
STROBE: Strengthening the reporting of observational studies in epidemiology
